# Sepsis in the Setting of Bilateral Submandibular Abscesses: A Case Report

**DOI:** 10.7759/cureus.114547

**Published:** 2026-08-14

**Authors:** Brooke D Johnson, Ava A Thielman, Shurouk Kattan Rahmani, Amaraja Kanitkar

**Affiliations:** 1 Emergency Medicine, University of Iowa, Iowa City, USA; 2 College of Osteopathic Medicine, A.T. Still University Kirksville College of Osteopathic Medicine, Kirksville, USA; 3 Internal Medicine, Henry Ford Warren Hospital, Warren, USA; 4 Pulmonary and Critical Care Medicine, Henry Ford Warren Hospital, Warren, USA

**Keywords:** bilateral submandibular abscesses, case report, critical care medicine, deep neck infections, emergency medicine, oral and maxillofacial surgery, sepsis, submandibular abscesses

## Abstract

Submandibular abscesses, a type of polymicrobial deep neck infection (DNI), are an otolaryngologic emergency due to the risk of airway compromise and septic shock. Unilateral submandibular abscesses are common and are often the result of odontogenic infections, while comorbidities that weaken the immune system, such as diabetes mellitus or human immunodeficiency virus (HIV), can exacerbate the infection. Bilateral submandibular abscesses are rare with limited cases reported in the literature, and are often associated with severe or systemic infections. This case report highlights the importance of a thorough physical examination and rapid intervention when evaluating a patient with suspected sepsis, presenting with nonspecific symptoms in the setting of multiple comorbidities. Rapid identification and appropriate management of a DNI such as bilateral submandibular abscesses is critical to preventing deterioration, including progression to sepsis, osteomyelitis, airway compromise, and death.

## Introduction

Deep neck infections (DNIs) are an otolaryngologic emergency that requires urgent medical intervention due to their rapid progression and life-threatening complications. Infections are polymicrobial and most commonly result from prior history of tonsillitis, pharyngitis, dental procedures, odontogenic sources, intravenous (IV) drug use, or surgery to the head and neck region [1]. DNIs arise within the potential spaces of the deep cervical neck layers, including the submandibular, retropharyngeal, and parapharyngeal spaces [2]. Infections often begin as cellulitis of the loose, areolar connective tissue and can evolve into abscesses. In some patients, abscesses can develop rapidly, within 48 hours [3]. Without intervention, possible complications include sepsis, Lemierre disease, mediastinitis, carotid artery aneurysm, necrotizing cervical fasciitis, and death from airway obstruction [2].

Submandibular abscesses are a type of DNI that commonly presents with a collection of pus, fever, pain, and swelling [4,5]. Before widespread use of antibiotics, submandibular abscesses commonly were caused by tonsillitis [2]. While it is still the most common cause in children, odontogenic sources, such as dental infections, are frequently observed in adults [6]. Sialadenitis of the submandibular gland, trauma, DNIs, lymphadenitis, and immunocompromised states, such as HIV or diabetes mellitus, are other causes of submandibular abscesses. Increased incidences of periodontitis and gingivitis complications in diabetics with poor glycemic control elevate the risk of a DNI. Through oral mucosa lacerations or perforations, the normal flora of the body can form abscesses [6]. Unilateral submandibular abscesses are common, usually from an odontogenic infection, which causes severe localized pain and potential airway compromise. Meanwhile, bilateral submandibular abscesses are rare and have an increased likelihood of life-threatening complications. The first priority in diagnostic workup is airway assessment, followed by contrast-enhanced computed tomography (CT) if the airway is stable. Upon confirmation, treatment includes removal of the infected tooth, incision and drainage (I&D) of the abscess, and broad-spectrum IV antibiotic therapy [6].

This case report details the clinical presentation, diagnostic workup, and the multidisciplinary management for a patient with bilateral submandibular abscesses. To our knowledge, bilateral involvement is rare, with only a limited number of cases reported in the literature and no well-validated population percentage established. This manuscript aims to emphasize the life-threatening nature of bilateral submandibular abscesses and highlight the importance of timely identification and appropriate treatment with a multidisciplinary approach.

This article was previously presented as a poster at the 2026 Annual Henry Ford Warren-Madison Heights Hospital Research Day on May 14, 2026.

## Case presentation

A 56-year-old female presented to the emergency department (ED) with several days of nausea, non-bilious, non-bloody emesis, non-bloody diarrhea, productive cough, chest pain, dyspnea, and generalized weakness. She reported odynophagia, dysphagia, weight loss, and a one-month history of progressive bilateral jaw and facial edema. She had a complex medical history, including type 2 diabetes mellitus, hypertension, hyperlipidemia, and obesity. Additionally, she had a history of epilepsy, bipolar disorder, coronary artery disease status post percutaneous coronary intervention with stent placement involving the left anterior descending coronary artery, cerebrovascular accident without residual deficits, hypothyroidism, and tobacco use disorder. She denied a fever, recent travel, sick contacts, or other surgeries.

Hemodynamically, the patient was tachycardic with a heart rate of 115 beats per minute (Table 1). Blood pressure, oxygen saturation, and temperature were within normal limits (Table 1). On physical examination, the patient had bilateral, diffuse facial and submandibular edema with tenderness to palpation over the left mandible. Intraoral examination demonstrated trismus, purulent drainage from the right mandibular vestibule, and carious dentition.

**Table 1:** Initial vital signs.

Vital Signs	Result	Unit	Reference Range
Temperature	98.1	°F	95.9–98.6 °F
Heart Rate	115	beats/min	60–100 beats/min
Respiratory Rate	16	breaths/min	14–20 breaths/min
Systolic Blood Pressure	107	mmHg	90–140 mmHg
Diastolic Blood Pressure	76	mmHg	60–90 mmHg
Oxygen Saturation	98	%	≥95%

Laboratory studies revealed leukocytosis with a white blood cell (WBC) count of 13.86 K/mcL and hypokalemia requiring correction (Table 2). The initial high-sensitivity troponin was elevated at 54 ng/L, with a repeat value of 65 ng/L, consistent with demand ischemia in the setting of suspected sepsis (Table 2). Serum lactate was less than 2 mmol/L, indicating no severe sepsis or septic shock (Table 2). Given the presence of leukocytosis, tachycardia, and clinical suspicion for infection, the patient fulfilled sepsis criteria, and empiric IV ampicillin-sulbactam (Unasyn) was initiated for broad-spectrum coverage of Gram-positive, Gram-negative, and anaerobic bacteria.

**Table 2:** Initial laboratory results.

Laboratory Test	Result	Unit	Reference Range
White blood cell count	13.86	K/mcL	4.0–11.0 K/mcL
Hemoglobin	11.6	gm/dL	12.0–16.0 gm/dL
Platelet count	398	K/mcL	150–400 K/mcL
Sodium	135	mmol/L	135–145 mmol/L
Potassium	2.3	mmol/L	3.5–5.2 mmol/L
Chloride	97	mmol/L	98–109 mmol/L
Bicarbonate	23	mmol/L	23–34 mmol/L
Blood urea nitrogen	27	mg/dL	8–20 mg/dL
Creatinine	0.88	mg/dL	0.7-1.20 mg/dL
Glucose	108	mg/dL	70–200 mg/dL
Aspartate aminotransferase	13	U/L	0-35 U/L
Alanine aminotransferase	<5	U/L	0-40 U/L
Lactic acid	1.5	mmol/L	0.5-2 mmol/L
High-sensitivity troponins	54; 65	ng/L	<10 ng/L

**Figure 1:**
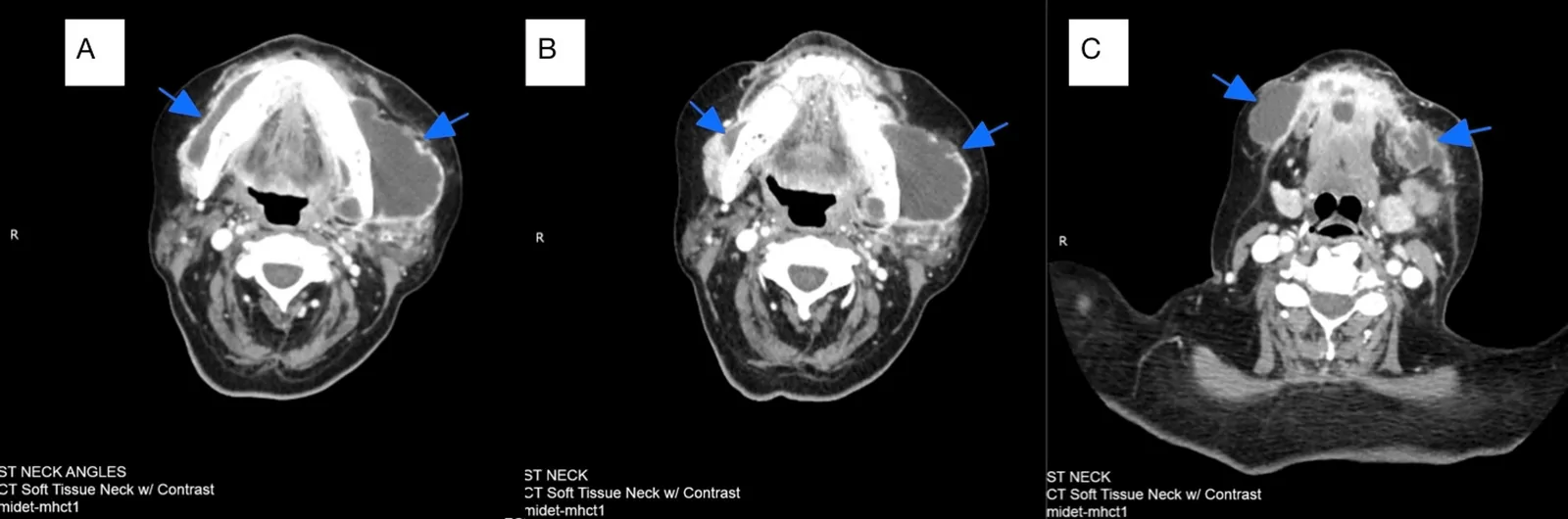
Axial contrast CT of the soft tissue neck and maxillofacial shows bilateral submandibular abscesses formation with suspected mandibular osteomyelitis. Panels A, B, and C demonstrate large complex bilateral submandibular fluid collections (blue arrows).

Chest radiograph showed no acute cardiopulmonary abnormalities. CT of the soft tissue neck and maxillofacial demonstrated 6.8 cm x 5.2 cm left and 5.4 cm x 1.2 cm right complex bilateral submandibular fluid collections consistent with abscess formation, non-displaced fracture of the inferior mandibular symphysis, and noted possible mandibular osteomyelitis (Figure 1). Electrocardiography showed normal sinus rhythm and no evidence of acute ST-elevation myocardial infarction with a markedly prolonged QTc interval of 715 ms.

Given concern for sepsis secondary to bilateral submandibular abscesses and the potential risk of airway compromise, the patient was admitted to the intensive care unit (ICU), and oral and maxillofacial surgery (OMFS) was consulted. After correction of electrolyte disturbances, particularly hypokalemia, the patient was brought to the operating room (OR) for I&D of the bilateral submandibular abscesses, extraction of affected teeth, and a biopsy of the anterior mandible. The procedure was complicated by concerns for potential airway obstruction due to the large abscesses, and the patient was electively intubated for airway protection. Intraoperative cultures of the abscess fluid were obtained, and soft tissue and bone specimens were sent to pathology for analysis. Bilateral neck incisions were made below the mandible to drain loculated infections. Two Penrose drains were placed on each side.

Post-operatively, the patient was closely monitored in the ICU. Cultures of the abscess fluid grew Fusobacterium nucleatum, which was susceptible to the ongoing ampicillin-sulbactam therapy. The pathology results of the anterior mandible biopsy demonstrated acute and chronic osteomyelitis, prompting a prolonged six-week course of IV antibiotic therapy.

**Figure 2:**
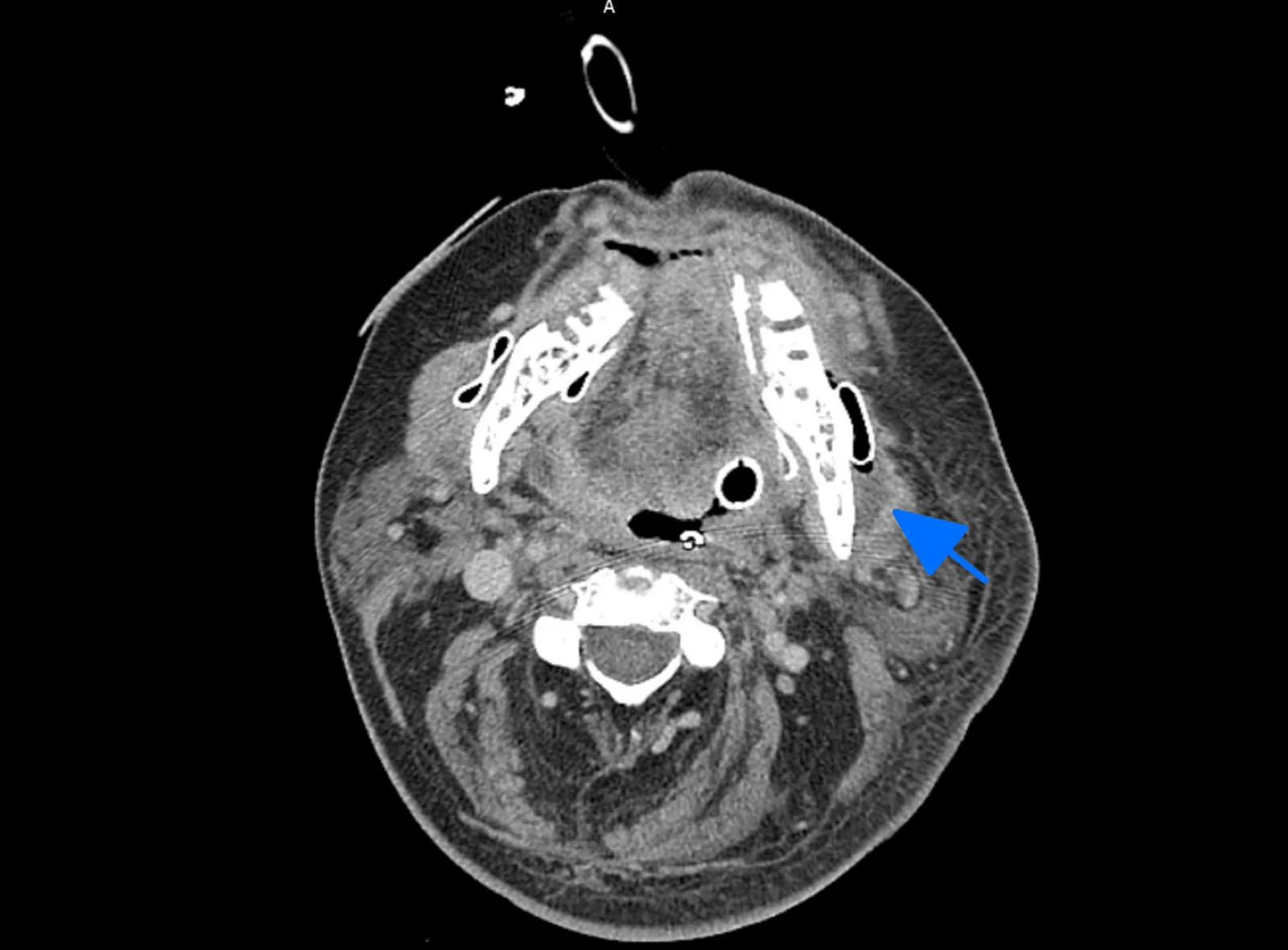
Post-operative axial contrast CT of the maxillofacial showing a right parasymphysis fracture with a remnant abscess in the left masticator space (blue arrow).

The patient’s condition gradually improved, and she was successfully extubated and transferred to a general medicine floor. A post-operative CT maxillofacial with contrast demonstrated lytic changes and a pathological right parasymphysis fracture with a small remnant abscess in the left masticator space (Figure 2). Given the patient’s continued clinical improvement, OMFS recommended no acute surgical intervention for the remnant abscess. She was discharged with plans to complete a prolonged course of IV antibiotics and to follow up outpatient with infectious disease and OMFS for a possible mandibular resection and reconstruction.

## Discussion

DNIs include submandibular abscesses, Ludwig’s angina, and peritonsillar abscesses. In a retrospective study examining 270 patients with a DNI, Ludwig’s angina was the most common (17.78%), followed by submandibular abscesses (13.33%) and peritonsillar abscesses (13.33%) [4]. Prior to the development of antibiotics, most DNIs were from tonsillitis; however, now the majority of adult cases are from odontogenic sources, while pediatric cases are more commonly from pharyngeal and tonsillar infections [2]. Our patient presented with bilateral submandibular abscesses in the setting of non-restorable and heavily decayed teeth, with underlying osteomyelitis prompting a full arch extraction.

While our patient had Fusobacterium nucleatum cultured, Streptococcus species are the most commonly isolated organisms in DNIs, accounting for 43.33% of cases [4]. Among the 270 patients studied, Streptococcus pyogenes was the most common pathogen (30.37%), followed by Staphylococcus aureus (22.97%), and Streptococcus viridans (12.96%) [4]. In another study, although Streptococcus species remained the most frequently isolated organisms, Bacteroides and Peptostreptococcus species were the most common anaerobic pathogens identified in cultures [2]. In patients with Lemierre disease, a clinically significant complication from a DNI, Fusobacterium necrophorum was the most common isolated organism [7]. This condition consists of septic thrombophlebitis of the internal jugular vein, consequently forming abscesses in the lungs from septic emboli. In diabetic patients, Klebsiella species are most frequently isolated [2]. In our patient, additional imaging was not pursued, as clinical features did not indicate thrombophlebitis.

Risk factors for the development of a DNI include smoking, alcohol consumption, and chewing tobacco. Diabetes mellitus and hypertension are the most common comorbidities associated with these patients. Pregnancy is another risk factor, as immune suppression during pregnancy may predispose individuals to infection [2]. Males are most commonly diagnosed with DNIs, accounting for approximately 57% of cases [4].

The diagnostic workup of a DNI includes imaging studies. While ultrasound is commonly readily available and inexpensive, it lacks the ability to detect small or deep infections. Contrast-enhanced CT is considered the gold standard for diagnosis [2]. The presence of air bubbles with fluid accumulation on CT increases the likelihood of abscess detection. Laboratory studies, including total WBC count, total neutrophil count, and lymphocyte count, may be unreliable when used alone to rule out DNI [3]. In a study by Boscolo-Rizzo et al., 365 patients with a DNI were examined. The authors found that the WBC was elevated in 46.8% of cases and normal in 52.6% [3]. The neutrophil counts were elevated in 56.4% and normal in 42.3% of cases, while lymphocyte counts were decreased in only 24.9% of cases and normal in 73.4% of cases [3]. These findings emphasize the importance of imaging in the diagnosis of a DNI.

Management of DNIs includes removal of the infected teeth, I&D, and IV antibiotics. In a study comparing intraoral and transcervical drainage approaches, there was no difference in hospitalization duration; however, the intraoral approach was associated with significantly shorter operative times and improved cosmetic outcomes [8]. In our patient, given the abscess extension to the bilateral masseteric and pterygomandibular spaces, a transcervical approach was chosen, which necessitated adequate external drainage. Anaerobic coverage with clindamycin or metronidazole is recommended in conjunction with a penicillin combined with a β-lactamase inhibitor, such as amoxicillin or ticarcillin with clavulanic acid [2]. Alternatively, β-lactamase-resistant antibiotics such as cefuroxime, cefoxitin, meropenem, or imipenem are acceptable options. For odontogenic causes of DNIs, anaerobic coverage is more important than for tonsillopharyngitis-related infections [2]. In diabetic patients, gentamicin should be added due to increased resistance of Klebsiella species to clindamycin. Blood glucose levels in these patients must be closely monitored to prevent complications and progression of the infection [2].

Significant neck and oropharyngeal edema may make endotracheal intubation under general anesthesia difficult due to limited neck extension. In such cases, tracheostomy is considered the gold standard for airway management; however, it carries a risk of spontaneous abscess rupture [2]. To minimize complications, fiberoptic intubation is recommended.

This patient's bilateral submandibular abscesses are rare because the compartmental anatomy of the submandibular functions to prevent crossover of infections. Bilateral involvement indicates a more advanced or aggressive disease process, often reflecting rapid spread across midline fascial planes. Fusobacterium necrophorum has a higher capacity for invasive spread and can lead to osteomyelitis formation, as seen in this patient, that most commonly presents through contiguous expansion of the infection to bone from adjacent soft tissue [7]. Given that abscess formation can occur within 48 hours, bilateral involvement may lead to rapid clinical deterioration. In such cases, the risk of airway obstruction - one of the leading causes of mortality in DNIs - is significantly increased. This possible complication further emphasizes the importance of early imaging, timely surgical intervention, and continuous airway assessment and monitoring.

Limitations of this case report include the unknown long-term follow-up of this patient, which precludes assessment of outcomes and treatment response. Nevertheless, this manuscript strengthens the limited current literature on the rare bilateral presentation of submandibular abscesses. Additionally, this case report emphasizes the importance of early recognition, thorough evaluation, and multidisciplinary management in accordance with current guidelines to prevent sepsis and airway compromise, as seen in this patient.

Future research should continue to focus on improving early detection of DNIs to reduce the risk of life-threatening complications, optimizing management strategies, and enhancing patient education to promote earlier presentation to the ED for treatment. Strengthening the existing literature on rare presentations, such as bilateral submandibular abscesses, and on the complications and management challenges associated with delayed diagnosis may further support timely recognition and improve clinical outcomes.

## Conclusions

Sepsis is a clinical syndrome that can originate from nearly any body system, but DNIs, like submandibular abscesses, are often overlooked in initial assessments. This case report emphasizes the importance of medical providers to consider less common sources of infection, especially in patients with risk factors such as poor dental hygiene, diabetes mellitus, or immunosuppression. Given the patient’s nonspecific complaints, including nausea, vomiting, and generalized weakness in the setting of suspected sepsis, a thorough physical exam was necessary, which included an intraoral examination and palpation of the neck. Timely identification of a DNI is critical in initiating appropriate management and preventing rapid deterioration, which could include sepsis, osteomyelitis, airway compromise, and death.
